# Advances in Staging of schizophrenia. Development of an empirical staging model for schizophrenia.

**DOI:** 10.1192/j.eurpsy.2023.32

**Published:** 2023-07-19

**Authors:** P. Garcia-Portilla

**Affiliations:** ^1^Psychiatry, Universidad de Oviedo; ^2^ISPA; ^3^CIBERSAM, Oviedo, Spain

## Abstract

**Abstract:**

After a short review of the state of the art of clinical staging in schizophrenia, I will present a recently developed empirical staging model.

**Methods:**

Two hundred twelve stable outpatients with schizophrenia from Oviedo (Spain) were assessed with: an ad hoc questionnaire (demographic, clinical information); psychopathology: PANSS, CDS, OSQ, CGI-S; functioning: PSP; cognition: MATRICS; lab tests: C-Reactive Protein, IL-1RA, IL-6, Platelet/Lymphocyte (PLR), Neutrophil/Lymphocyte (NLR), and Monocyte/Lymphocyte (MLR) ratios.

An ad hoc genetic algorithm (GA) was developed to select those variables showing the best performance for patients’ CGI classification. The objective function of the GA maximizes the individual’s correct classification of a support vector machines (SVM) model that employs as input variables those given by the GA. Models’ performance was assessed with the help of 3-fold cross-validation, and this process was repeated 10,000 times for each one of the models evaluated. Once developed, we used the ANOVA test (Duncan’s post-hoc) for all the variables included in the model to demonstrate its construct validity.

**Results:**

Our model included the following variables: positive, negative, depressive, and general psychopathological symptoms, processing speed, visual learning, social cognition, and real-world functioning. Its classification accuracy is 64.54% (SD=4.83%) with a specificity and sensitivity of 0.85 and 0.63. The external validity of the new model is being tested using a French sample from the FACE-SZ (FondaMental Advanced Centers of Expertise-Schizophrenia) cohort.

**Conclusions:**

We developed an SVM model including psychopathological, cognitive, and functional variables.

Our model demonstrated good construct validity since all the variables included behaved as expected. That is, they score significantly worse as the patients’ severity increases. Besides, it showed good accuracy, specificity, and sensitivity classification properties.

Using staging models in daily clinical practice will help clinicians to better.

**Disclosure of Interest:**

None Declared

